# Nomogram to predict 6-month mortality in acute ischemic stroke patients treated with endovascular treatment

**DOI:** 10.3389/fneur.2023.1330959

**Published:** 2024-01-05

**Authors:** Rui Wen, Miaoran Wang, Wei Bian, Haoyue Zhu, Ying Xiao, Qian He, Yu Wang, Xiaoqing Liu, Yangdi Shi, Zhe Hong, Bing Xu

**Affiliations:** ^1^Shenyang Tenth People’s Hospital, Shenyang, China; ^2^Affiliated Central Hospital of Shenyang Medical College, Shenyang Medical College, Shenyang, China; ^3^Shenyang First People’s Hospital, Shenyang Medical College, Shenyang, China

**Keywords:** nomogram, prediction, mortality, ischemic stroke, endovascular treatment

## Abstract

**Background:**

Acute Ischemic Stroke (AIS) presents significant challenges in evaluating the effectiveness of Endovascular Treatment (EVT). This study develops a novel prognostic model to predict 6-month mortality post-EVT, aiding in identifying patients likely to benefit less from this intervention, thus enhancing therapeutic decision-making.

**Methods:**

We employed a cohort of AIS patients from Shenyang First People’s Hospital, serving as the Validation set, to develop our model. LASSO regression was used for feature selection, followed by logistic regression to create a prognostic nomogram for predicting 6-month mortality post-EVT. The model’s performance was validated using a dataset from PLA Northern Theater Command General Hospital, assessing discriminative ability (C-index), calibration (calibration plot), and clinical utility (decision curve analysis). Statistical significance was set at *p* < 0.05.

**Results:**

The development cohort consisted of 219 patients. Six key predictors of 6-month mortality were identified: “Lack of Exercise” (OR, 4.792; 95% CI, 1.731–13.269), “Initial TICI Score 1” (OR, 1.334; 95% CI, 0.628–2.836), “MRS Score 5” (OR, 1.688; 95% CI, 0.754–3.78), “Neutrophil Percentage” (OR, 1.08; 95% CI, 1.042–1.121), “Onset Blood Sugar” (OR, 1.119; 95% CI, 1.007–1.245), and “Onset NIHSS Score” (OR, 1.074; 95% CI, 1.029–1.121). The nomogram demonstrated a high predictive capability with a C-index of 0.872 (95% CI, 0.830–0.911) in the development set and 0.830 (95% CI, 0.726–0.920) in the validation set.

**Conclusion:**

Our nomogram, incorporating factors such as Lack of Exercise, Initial TICI Score 1, MRS Score 5, Neutrophil Percentage, Onset Blood Sugar, and Onset NIHSS Score, provides a valuable tool for predicting 6-month mortality in AIS patients post-EVT. It offers potential to refine early clinical decision-making and optimize patient outcomes, reflecting a shift toward more individualized patient care.

## Introduction

Globally, ischemic stroke is one of the leading causes of death and disability (1). Acute Ischemic Stroke (AIS), a predominant subtype of acute stroke, manifests from cerebral ischemia due to vascular thrombosis (2). The primary etiologies for AIS include atherosclerotic stenosis, thrombosis, and embolic events. Given its high morbidity, mortality, and recurrence rates, timely intervention for AIS is imperative (3).

Endovascular Treatment (EVT) has been recognized as a pivotal therapeutic strategy for AIS, offering tangible clinical benefits despite associated financial implications (4–7). However, a significant clinical challenge is the phenomenon of “futile reperfusion,” where 19%–43% of EVT recipients achieve vascular reperfusion but do not experience corresponding functional recovery (1–3, 5–7). This underscores the importance of understanding long-term mortality trajectories post-EVT, especially in high-risk demographics such as the elderly. Current evidence, including findings from the Third International Stroke Trial (IST-3), suggests that age should not be the sole determinant for excluding patients from recanalization strategies (8, 9). Within this context, a 6-month mortality metric post-EVT is invaluable, providing clinicians with a comprehensive understanding of treatment outcomes, ensuring evidence-based clinical decisions (8–10).

While successful endovascular reperfusion brings promise, it does not universally guarantee a favorable prognosis. Post-EVT, a significant fraction of AIS patients remain vulnerable to comorbidities, treatment ineffectiveness, and heightened mortality risks (11–13). This underscores a challenge; a significant proportion of patients undergoing EVT might not attain survival.

Against this backdrop, the pressing need arises for advanced prognosis tools. The nomogram, a graphical instrument, has been widely adopted in diverse medical fields, such as surgery, cancer, and myocardial infarction. It allows swift prognosis predictions based on selected clinical and laboratory parameters (14–17). Intriguingly, while several nomograms geared toward predicting unfavorable outcomes in AIS patients post-EVT are available (18–21), only a sparse few focus on mortality, with an even rarer emphasis on a 6-month prognostic timeframe (21, 22).

Addressing this discernible void, our research diligently seeks to intertwine various pre-treatment indicators, such as demographic and clinical factors, with the utmost aim to construct a novel prognostic model. This model is not solely crafted to predict the probability of 6-month mortality following EVT in AIS patients, but is also crucially intended to identify those individuals who might derive lesser benefit from EVT, potentially guiding clinicians in their therapeutic decision-making process.

## Methods

### Study design and participants

This research is a retrospective cohort study, grounded on two distinct cohorts. It meticulously compiles data from AIS patients who underwent EVT. These cohorts are divided into training and validation sets, a strategy that significantly bolsters the robustness and credibility of the nomogram developed from this study. The training set was retrospectively curated from Shenyang First People’s Hospital, integrating data from 331 AIS patients treated with EVT spanning from October 2017 to April 2023. Concurrently, the validation set comprised of 103 AIS patients, extracted from the PLA (People’s Liberation Army) Northern Theater Command General Hospital, who underwent EVT between March 2019 and December 2021. These two datasets, derived from diverse patient pools and different time frames, afforded a comprehensive platform for the development and subsequent validation of the prognostic nomogram, ensuring a meticulous and thorough analytical approach toward predicting 6-month mortality post-EVT in AIS patients. The data for both cohorts were obtained from the Shenyang Stroke Emergency Map data system, an initiative spearheaded by the Shenyang First People’s Hospital. Given that this centralized system was responsible for data collection and management, ethical approval for the study was sought and granted by the Research Ethics Committee of Shenyang First People’s Hospital (Number: 2023SYKYP251), encompassing the data used from both centers.

Patients were included in development cohort if all the following conditions were met: (1) were treated with EVT; (2) were aged ≥18 years; (3) were followed up 6 months; (4) cause of death was related to AIS; (5) had occlusion at the internal carotid artery, middle cerebral artery, basilar artery, posterior cerebral artery, or vertebral artery confirmed by computed tomographic angiography, magnetic resonance angiography, or digital subtracted angiography. Patients with malignant tumor, autoimmune disease, severe renal insufficiency, hepatic disease, or heart failure were excluded.

### Data collection

Data compilation was executed through a diligently crafted case-report form, encapsulating both demographic and clinical variables to weave a comprehensive analytical framework for this study. Primary demographic variables, specifically age and gender, were noted alongside an assortment of clinically pertinent and lifestyle-oriented variables, such as weight and smoking status. Historical data regarding previous strokes and existing medical conditions, in conjunction with stroke subtypes, and respective scoring via the NIHSS upon onset, were documented to underpin nuanced clinical insights.

Procedural intricacies and specifics regarding stroke onset and treatment, such as MRS Score and Thrombolysis status, were similarly recorded. An array of laboratory parameters was gathered, providing a detailed vista of the patients’ physiological state, while the 6-month mortality status was identified as the primary outcome variable. Follow-up evaluations utilizing the modified Rankin Scale at the 6-month mark were systematically conducted across all participating centers, executed either through telephonic consultations or outpatient visits, ensuring meticulous recording and assessment of mortality.

After the meticulous recording and assessment of mortality, we consolidated the data from the two participating centers, ensuring a comprehensive dataset for subsequent analysis. However, despite our systematic data collection approach, some missing values were observed, necessitating the adoption of a strategic imputation method to uphold the validity of our analysis. We proceeded with the creation of 20 imputed datasets. For the purpose of aggregation, the imputed values across these datasets were averaged, with categorical variables determined by mode. Post-imputation, a thorough analysis of each dataset ensued. To consolidate the findings, Rubin’s rules were meticulously applied, yielding a harmonized output. To corroborate the robustness of our imputation technique, a sensitivity analysis juxtaposing the imputed and the original data sets was performed, the details of which have been annexed.

### Statistical analysis

Statistical analysis was performed using R statistical software (version 4.1.2;R Foundation for Statistical Computing, Vienna, Austria). Categorical variables were expressed as *n* (%) and continuous variables as means (SD) or medians (interquartile range). Differences in baseline characteristics between groups were analyzed using independent sample *t* tests, Mann–Whitney *U* tests for continuous variables, and the chi-squared test or Fisher’s exact test for categorical variables, as appropriate.

In the development of the nomogram, we initially employed the Least Absolute Shrinkage and Selection Operator (LASSO) regression method, which is notably effective in handling high-dimensional data, thereby facilitating the selection of relevant variables. These variables were then applied in logistic regression modeling using the training set. The developed nomogram was subsequently subjected to rigorous external validation using a separate validation set. The model’s discriminative capacity was quantified by the concordance index (C-index), and a calibration plot, constructed from 10,000 bootstrap resamples, was used to assess the congruence between the predicted mortality outcomes and those observed in the validation set.

Decision curve analysis was used to evaluate the validity of the nomogram. Detailed descriptions of the decision curve analysis were previously reported (23). Results were considered statistically significant at *p* < 0.05.

## Results

**Figure 1:**
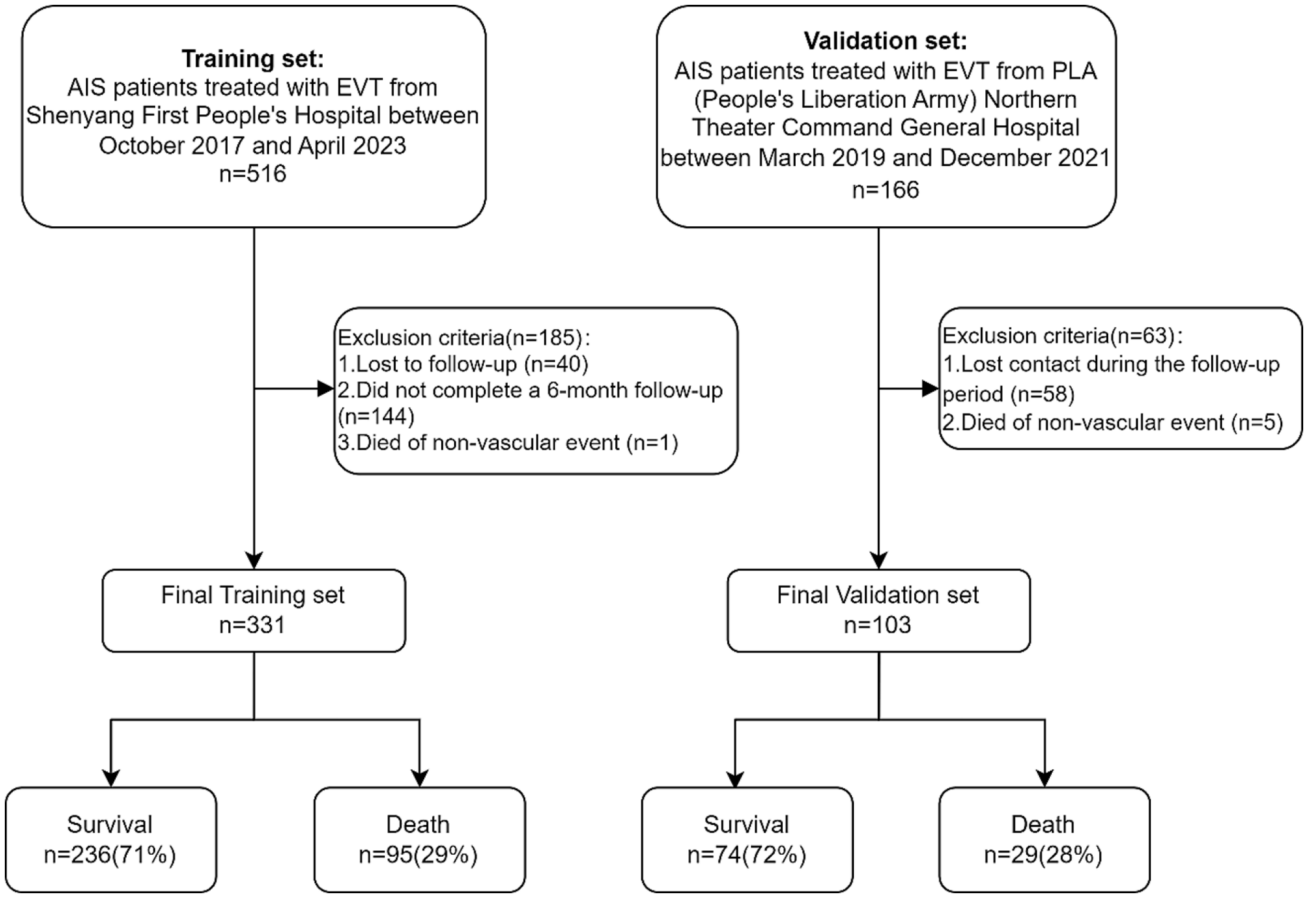
Flow chart outlining the patient inclusion process. AIS, acute ischemic stroke; EVT, endovascular therapy.

The flow chart outlining the patient inclusion process is shown in Figure 1. In the study, the development and validation cohorts comprised 331 (median age 66 years; 28.40% female) and 103 (median age 67 years; 31.07% female) eligible patients, respectively. No marked discrepancies in mortality were observed between these groups (29.70% vs. 28.16%; *p* > 0.9). Significant differences were detected between the cohorts in factors such as smoking (38.67% in the development cohort vs. 24.27% in the validation cohort; *p* = 0.008), history of ischemic stroke (34.74% vs. 19.42%, *p* = 0.006), onset NIHSS score (12 vs. 15, *p* = 0.005), and initial TICI score (*p* < 0.001). Furthermore, the study identified disparities in laboratory results like neutrophil count, direct bilirubin, white blood cell count, blood urea, ALT, total bilirubin, creatinine, uric acid, blood sugar at onset, homocysteine, albumin-globulin ratio, cholesterol, AST, body temperature, and heart rate between the two cohorts (all *p* < 0.05). Detailed baseline characteristics of the development and validation cohorts can be found in Table 1.

**Table 1:** Demographics and clinical characteristics of the training and validation set.

Variable		Validation set (*n* = 103)	Training set (*n* = 331)	*p*-value
Age (years)		67 (59, 74)	66 (60, 73)	0.700
Gender (% females)		32 (31.07%)	94 (28.40%)	0.600
Weight (kg)		71 (67, 73)	70 (63, 75)	>0.900
Smoking		25 (24.27%)	128 (38.67%)	0.008
Drinking		19 (18.45%)	69 (20.85%)	0.600
Lack of exercise		5 (4.85%)	28 (8.46%)	0.200
Hx of hemorrhagic stroke		3 (2.91%)	12 (3.63%)	>0.900
Hx of ischemic stroke		20 (19.42%)	115 (34.74%)	0.006
Daytime stroke		77 (74.76%)	241 (72.81%)	0.700
CC disease		89 (86.41%)	270 (81.57%)	0.300
Metabolic endocrine disease		39 (37.86%)	98 (29.61%)	0.130
Other diseases/surgery		10 (9.71%)	49 (14.80%)	0.140
Family Hx of CC disease		4 (3.88%)	12 (3.63%)	>0.900
PACI		54 (52.43%)	175 (52.87%)	>0.900
TACI		21 (20.39%)	78 (23.56%)	0.500
LACI		4 (3.88%)	10 (3.02%)	0.500
POCI		27 (26.21%)	71 (21.45%)	0.200
Onset NIHSS score		15 (11, 18)	12 (3, 20)	0.005
Onset MRS score				<0.001
	0	68 (66.02%)	0 (0.00%)	
	1	4 (3.88%)	63 (19.03%)	
	2	2 (1.94%)	30 (9.06%)	
	3	4 (3.88%)	22 (6.65%)	
	4	12 (11.65%)	102 (30.82%)	
	5	13 (12.62%)	114 (34.44%)	
Thrombolysis		36 (34.95%)	132 (39.88%)	0.400
Initial TICI score				<0.001
	0	93 (90.29%)	96 (29.00%)	
	1	4 (3.88%)	55 (16.62%)	
	2a	3 (2.91%)	126 (38.07%)	
	2b	1 (0.97%)	8 (2.42%)	
	3	2 (1.94%)	46 (13.90%)	
Post-Embolization Narrowing		4 (3.88%)	8 (2.42%)	0.500
GA		21 (20.39%)	33 (9.97%)	0.004
Neutrophil Count (x10^9/L)		7.4 (7.1, 8.1)	6.3 (4.4, 8.8)	<0.001
NA		8	36	
DB (mg/dL)		3.30 (3.02, 3.52)	3 (2.10, 3.90)	0.017
NA		14	42	
Platelet Count (x10^9/L)		30 (10, 184)	29 (11, 214)	0.200
NA		17	36	
SA (g/dL)		41.1 (40.2, 41.7)	41.1 (38.3, 44.0)	>0.900
NA		8	42	
WBC (x10^9/L)		9.58 (9, 10.09)	8.37 (6.61, 10.74)	<0.001
NA		8	36	
BU (mg/dL)		5.87 (5.56, 6.14)	5.26 (4.40, 6.54)	<0.001
NA		9	36	
Globulin		26.2 (25.5, 27.0)	25.8 (23.4, 28.5)	0.130
NA		9	41	
ALT (U/L)		21 (19, 23)	17 (13, 24)	<0.001
NA		10	33	
Neutrophil percentage		76 (75, 79)	75 (64, 85)	0.300
NA		14	36	
Hb (g/dL)		143 (131, 150)	136 (100, 161)	0.068
NA		14	36	
TB (mg/dL)		17.1 (16.2, 18.3)	15.4 (12.1, 20.0)	0.003
NA		8	42	
Creatinine (mg/dL)		74 (68, 78)	69 (58, 82)	0.010
NA		10	36	
UA (mg/dL)		333 (313, 351)	311 (257, 376)	0.017
NA		10	30	
Onset blood sugar (mg/dL)		7.42 (6.97, 7.96)	6.40 (5.40, 8.57)	<0.001
NA		6	16	
Hcy (μmol/L)		19 (17, 21)	16 (13, 21)	<0.001
NA		14	50	
Lymphocyte_Count		1.45 (1.30, 1.56)	1.42 (0.96, 1.93)	0.800
NA		9	36	
Total protein (g/dL)		67.4 (66.5, 68.3)	67.0 (63.4, 70.7)	0.500
NA		9	42	
RBC (x10^12/L)		4.34 (4.25, 4.50)	4.44 (4.02, 4.81)	0.120
NA		14	36	
Lymphocyte percentage		16 (14, 18)	16 (10, 27)	0.600
NA		17	36	
Albumin globulin ratio		2.05 (1.62, 2.27)	1.62 (1.42, 1.81)	<0.001
NA		9	41	
Cholesterol (mg/dL)		4.74 (4.60, 4.97)	4.55 (3.54, 5.53)	0.080
NA		14	51	
AST (U/L)		23 (22, 25)	20 (18, 26)	<0.001
NA		7	29	
SBP (mmHg)		148 (134, 166)	146 (134, 160)	0.800
DBP (mmHg)		86 (76, 91)	85 (80, 91)	0.500
Temperature (°C)		36.31 (36.30, 36.33)	36.40 (36.30, 36.50)	<0.001
Heart rate (bpm)		79 (77, 80)	76 (70, 80)	<0.001
6-month death		29 (28%)	95 (29%)	>0.900

Hx, History; CC disease, Cardiocerebral Disease; PACI, Partial Anterior Circulation Infarct; TACI, Total Anterior Circulation Infarct; LACI, Lacunar Infarct; POCI, Posterior Circulation Infarct; NIHSS, National Institutes of Health Stroke Scale; MRS, Modified Rankin Scale; TICI, Thrombolysis in Cerebral Infarction; GA, General Anesthesia; DB, Direct Bilirubin; SA, Serum Albumin; WBC, White Blood Cell; ALT, Alanine Aminotransferase; Hb, Hemoglobin; TB, Total Bilirubin; UA, Uric Acid; BU, Blood Urea; Hcy, Homocysteine; A/G Ratio, Albumin/Globulin Ratio; AST, Aspartate Aminotransferase; SBP, Systolic Blood Pressure; DBP, Diastolic Blood Pressure; Lack of Exercise, exercise less than 3 times a week, and less than 30 min each time; Metabolic Endocrine Disease, a group of conditions affecting the body’s metabolism, including diabetes, thyroid disorders (such as hypothyroidism or hyperthyroidism), and other gland-related diseases.

**Figure 2:**
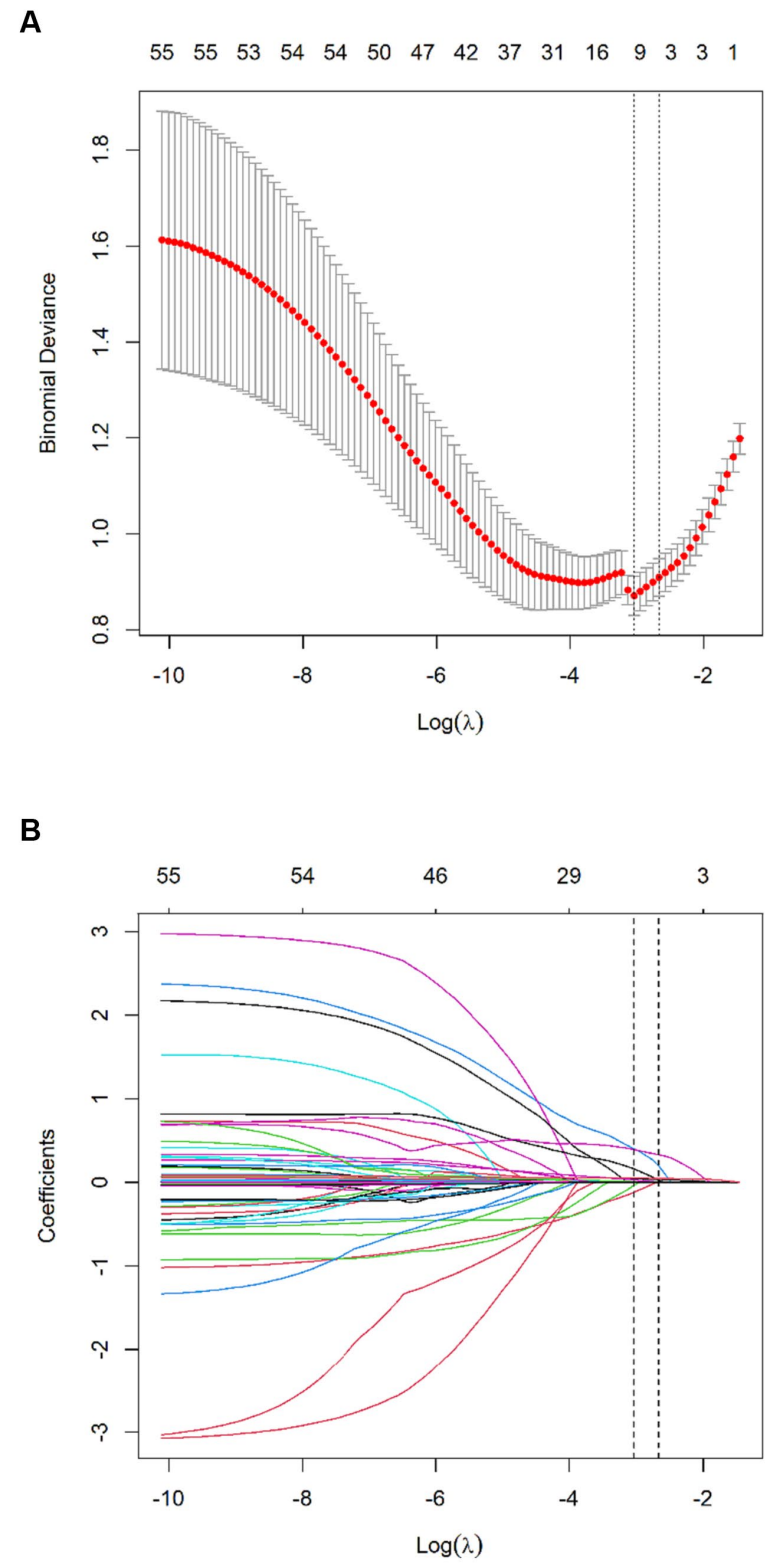
Texture feature selection using the least absolute shrinkage and selection operator (LASSO) binary logistic regression model. **(A)** Tuning parameter (λ) selection in the LASSO model used 10-fold cross-validation via minimum criteria. The Binomial Deviance was plotted vs. log (λ). Dotted vertical lines were drawn at the optimal values by using the minimum criteria and the 1 standard error of the minimum criteria (the 1-SE criteria). A λ value of 0.048, with log (λ), −3.034 was chosen (the minimum criteria) according to 10-fold cross-validation. **(B)** LASSO coefficient profiles of the 57 features. A coefficient profile plot was produced against the log (λ) sequence. Vertical line was drawn at the value selected using 10-fold cross-validation, where optimal λ resulted in 6 nonzero coefficients.

**Figure 3:**
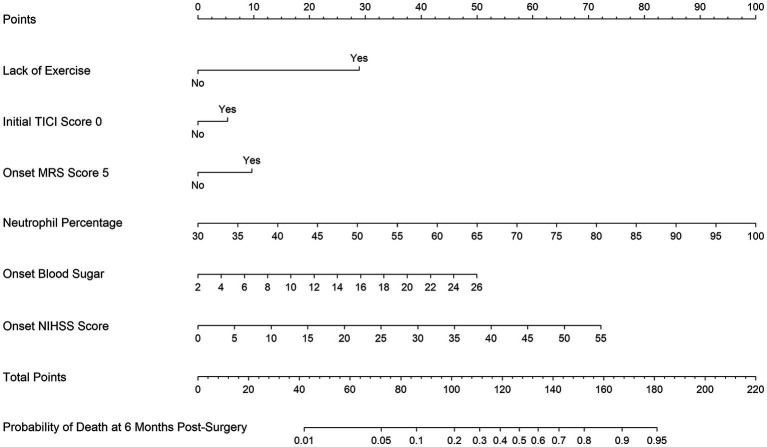
Nomogram for predicting the probability of 6-month mortality in Chinese acute ischemic stroke patients undergoing endovascular treatment based on Lack of Exercise, Initial TICI Score 1, MRS Score 5, Neutrophil Percentage, Onset Blood Sugar, and Onset NIHSS Score.

Of features, 57 features were reduced to 6 potential predictors on the basis of 331 patients in the training set (9.5:1 ratio; Figures 2A,B), and were features with nonzero coefficients in the LASSO logistic regression model. Utilizing the 6 predictors identified through LASSO logistic regression from the initial 57, a predictive model was subsequently developed, ensuring concise and reliable forecasting in our study context. Furthermore, a nomogram was established to visually represent the predictive model, aiding clinicians in conveniently calculating the probability of the outcome by aligning each variable’s value with its associated points (Figure 3). In the multivariate analysis of the training set (Table 2), six potential predictors were identified through the LASSO logistic regression model. Specifically, “Lack of Exercise” (OR, 4.792; 95% CI, 1.731–13.269; *p* = 0.003), “Initial TICI Score 1” (OR, 1.334; 95% CI, 0.628–2.836; *p* = 0.453), “MRS Score 5” (OR, 1.688; 95% CI, 0.754–3.78; *p* = 0.203), “Neutrophil Percentage” (OR, 1.08; 95% CI, 1.042–1.121; *p* < 0.001), “Onset Blood Sugar” (OR, 1.119; 95% CI, 1.007–1.245; *p* = 0.037), and “Onset NIHSS Score” (OR, 1.074; 95% CI, 1.029–1.121; *p* = 0.001) were evaluated. Notably, “Lack of Exercise,” “Onset Blood Sugar,” and “Onset NIHSS Score” were found to be statistically significant predictors, with *p*-values below the conventional 0.05 threshold. Conversely, despite “Initial TICI Score 1,” “MRS Score 5,” and “Neutrophil Percentage” not achieving statistical significance, they were retained in the model owing to their nonzero coefficients in the LASSO logistic regression model. Each variable uniquely influenced the model’s predictive capability, providing varied insights into the potential risk and protective factors pertinent to the outcome.

**Table 2:** Multivariate analysis of the training set.

Intercept and variable	β	Odds ratio (95% CI)	*P*
Intercept	−9.411		<0.001
Lack of exercise	1.567	4.792(1.731–13.269)	0.003
Initial TICI Score 1	0.289	1.334(0.628–2.836)	0.453
MRS Score 5	0.523	1.688(0.754–3.78)	0.203
Neutrophil percentage	0.077	1.08(1.042–1.121)	<0.001
Onset blood sugar	0.113	1.119(1.007–1.245)	0.037
Onset NIHSS score	0.071	1.074(1.029–1.121)	0.001

**Figure 4:**
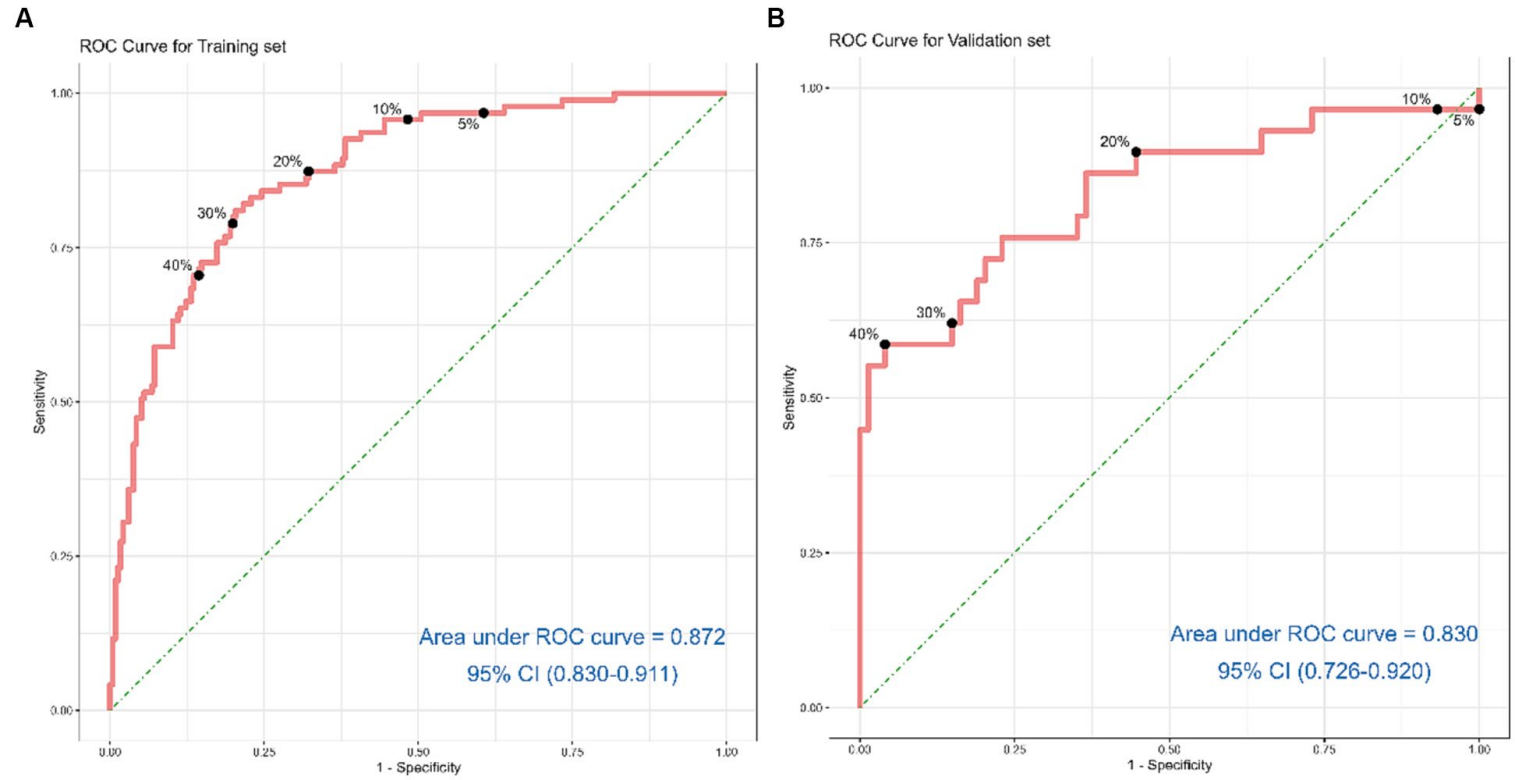
Receiver operating characteristic curves for risk of 6-month mortality in acute ischemic stroke patients treated with endovascular treatment. **(A)** shows the ROC Curve for the Training set. **(B)** depicts the ROC Curve for the Validation set. Sensitivity and specificity of several risk thresholds of the prediction model are plotted.

**Figure 5:**
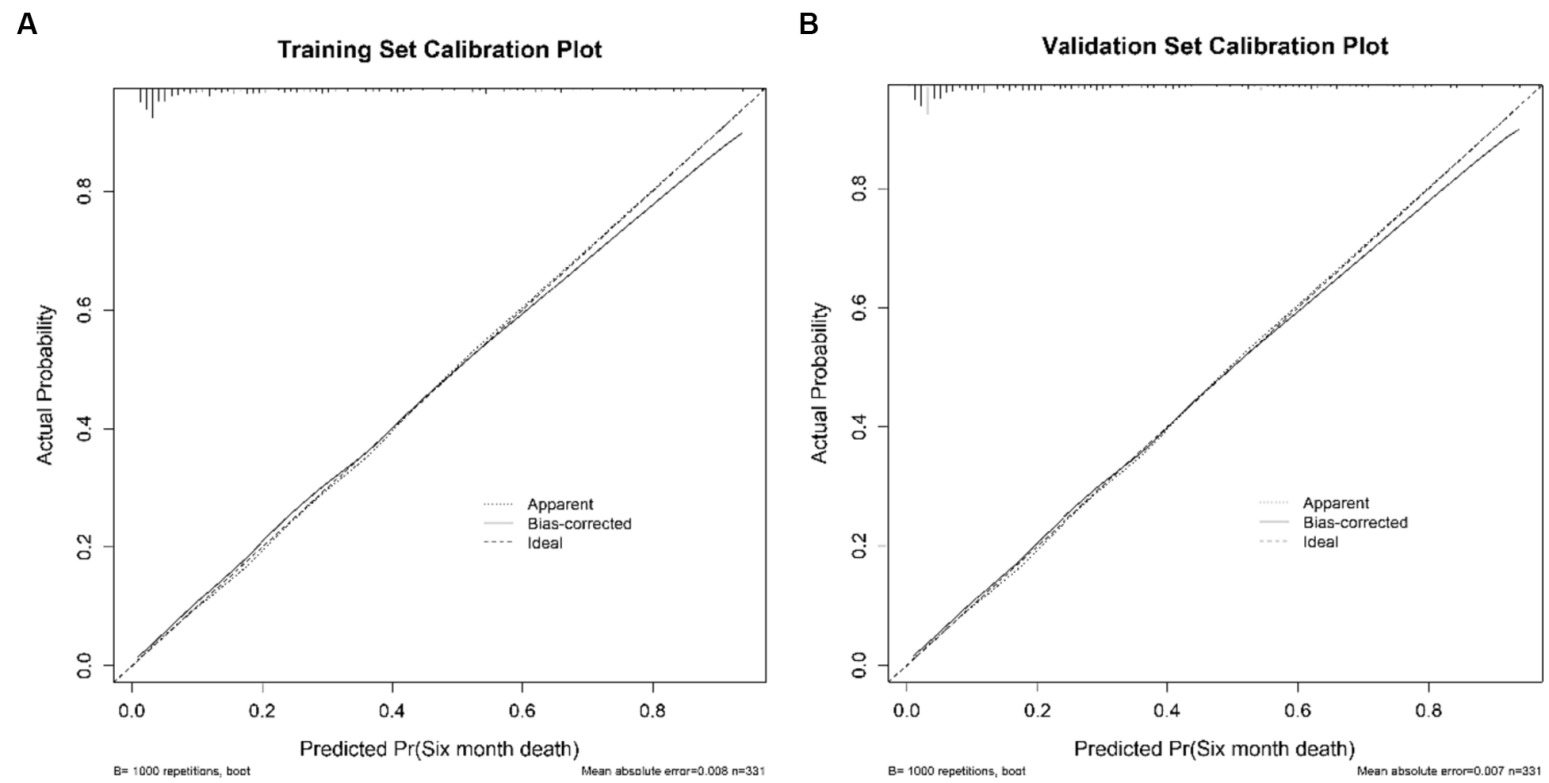
The calibration curves of the nomogram for predicting 6-month mortality of stroke patients treated with EVT. **(A)** presents the Calibration Plot for the Training Set. **(B)** shows the Calibration Plot for the Validation Set. The x-axis represents the predicted probability of unfavorable outcome calculated using the nomogram. The y-axis represents the actual rate of unfavorable outcome. The dashed line is the reference line where an ideal nomogram would lie, the dotted line represents the nomogram’s performance, and the solid line adjusts for any deviation from the nomogram.

Discrimination of the nomogram, when assessed on the training set, was measured by calculating the c-index, which was 0.872 (95% CI, 0.830–0.911), indicating good predictive power (Figure 4A). Similarly, evaluation on the validation set yielded a c-index of 0.830 (95% CI, 0.726–0.920), reflecting the nomogram’s consistent performance across different datasets (Figure 4B). Figure 5 shows a calibration plot, comparing the prediction of mortality between the nomogram prediction and actual observation on the same training set. The calibration plot revealed good predictive accuracy of the nomogram (Figure 5A). Furthermore, the model was externally validated using the test cohort with a c-index of 0.830 (95% CI, 0.726–0.920). Given that a c-index > 0.75 is generally considered to indicate reliable discrimination, this nomogram performed well using both the training and validation set. The satisfactory calibration of the nomogram was also confirmed by the validation set (Figure 5B).

**Figure 6:**
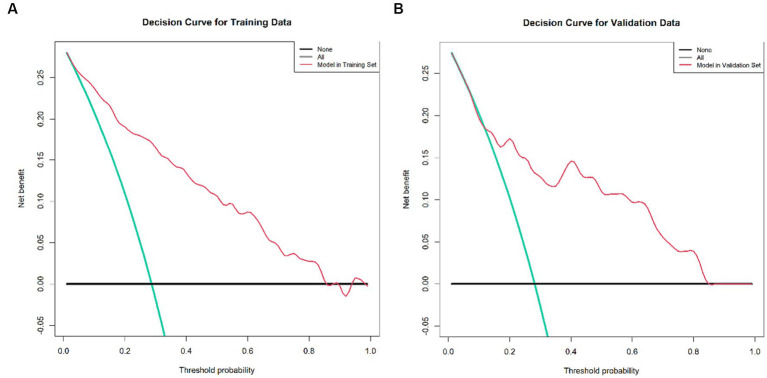
Decision curve analysis of the nomogram, encompassing both the training and validation set. **(A)** Decision Curve for the Training Data set, demonstrating the net benefit across various threshold probabilities. **(B)** Decision Curve for the Validation Data set, depicting corresponding net benefits. The x-axis illustrates the threshold probability while the y-axis measures the net benefit. The black, red, and green lines respectively represent the net benefit of universally treating all patients, treating no patients, and the nomogram’s application. This decision curve analysis method is employed to evaluate the prognostic value of nomogram strategies, with the developed nomogram specifically designed to assess the probability of post-EVT mortality among AIS patients. Notably, the quintessential goal of this nomogram is not merely to gauge post-EVT mortality probability but pivotally to distinguish patients who may derive marginal benefit from EVT. Consequently, it enables clinicians to identify and subsequently tailor therapeutic interventions for those at a high risk of post-EVT death—potentially involving additional procedures such as hematoma evacuation or decompressive craniectomy—while avoiding potentially non-beneficial interventions for those at lower risk. In the context of this study, the reference risk was calculated presuming a universal need for further treatment to prevent death, with a zero net benefit defining a scenario where no patients require additional intervention. The threshold probability is determined at the point where the anticipated benefit of further therapy aligns with the expected benefit of foregoing additional intervention. Consequently, the most favored model is the nomogram that yields the highest net benefit at any given threshold probability.

Decision curve analysis estimates the net benefit of a model by considering the difference between the numbers of true and false-positive results. This method is widely used to assess whether decisions assisted by a nomogram would improve patient outcomes. As shown in Figure 6, the decision curve analysis indicated that when the threshold probabilities ranged between 4% and 85% in the training set, and between 12% and 83% in the Validation set, the use of the nomogram to predict 3-month mortality provided greater net benefit than the “treat all” or “treat none” strategies, which indicates the clinical usefulness of the nomogram. For example, if the personal threshold probability of a patient is 40% (the patient would opt for treatment if the probability of mortality were >40%), then the net benefit is 0.134 in the training set and 0.146 in the test set.

## Discussion

In the present study, a meticulous nomogram was constructed, harnessing parameters such as Lack of Exercise, Initial TICI Score 1, MRS Score 5, Neutrophil Percentage, Onset Blood Sugar, and Onset NIHSS Score, to proficiently predict the 6-month mortality probability for anterior circulation stroke patients who underwent successful EVT. The exemplary discrimination and calibration of the nomogram were evident in the training cohort and corroborated through external validation. A distinctive merit of our model lies in its reliance on pre-treatment indicators, all of which are obtainable before initiating any therapeutic intervention. This not only enhances its clinical value but also furnishes healthcare providers, patients, and their families with a pragmatic reference, allowing for an informed decision-making process from an early stage.

In extant literature, various prognostic scores, such as the IER-START score (20), THRIVE score (24), and NAC score (25), have been proposed to predict clinical outcomes at 3 months post-mechanical thrombectomy for stroke patients. While these scores do provide valuable prognostic information to some extent, their point-based risk scoring systems—which hinge upon predetermined cutoff values for discrete and continuous variables like age, baseline NIHSS score, blood glucose level, and creatinine—might not fully capitalize on within-category information, thereby potentially compromising predictive accuracy. Moreover, despite these studies offering valuable insights, the predominant focus on a 3-month prognostic timeframe somewhat limits our understanding of patients’ longer-term recovery trajectories (18, 19, 21). Diverging from these earlier investigations, our study encompasses not only the method of mechanical thrombectomy but also a broader array of intravascular treatments, such as stent installation, and crucially, adopts a 6-month point for prognostic observation. This research direction aims not merely to offer a more expansive perspective to observe and evaluate patients’ medium-to-long-term trajectories post-intravascular treatment but also fundamentally seeks to discuss and dialectically analyze the genuine necessity and efficacy of these intravascular treatments for patients with high mortality risk within 6 months. In the context of current clinical practice, the principle of avoiding over-medicalization has gradually gained recognition. Thus, our research also aims to discern whether every kind of intravascular treatment—especially those more invasive ones, like stent installation—is indeed necessary and value-concordant for those patients with higher mortality risk within 6 months. This could not only aid clinicians in making more precise and rational treatment decisions when confronted with varying patient scenarios but also proactively enhance patient safety and medical efficacy to a certain extent.

In our quest to identify the pivotal determinants predicting 6-month mortality following intravascular treatment in ischemic stroke patients, our model illuminated a series of key variables. One such variable, Lack of Exercise, was underscored as a considerable precursor to unfavorable outcomes. This resonates with previous findings that have delineated the crucial role of pre-stroke physical activity in influencing post-intervention prognosis (26). Lack of exercise is especially significant in patients with vascular risk factors, particularly those diagnosed with metabolic syndrome (27). If these patients do not engage in regular physical activity, the progression of arteriosclerosis accelerates. Coupled with the poor management of other vascular risk factors, such as hypertension and diabetes, this can further intensify vascular damage and pathology (28). Over time, the severity of vascular diseases can worsen, increasing the risk of stroke and other cardiovascular events. Similarly, an initial TICI score of 1, signaling minimal or absent reperfusion, was identified as a harbinger of heightened mortality risk (29, 30), underscoring the indispensable value of successful reperfusion in determining post-treatment trajectories. The MRS score of 5, indicating severe disability at the outset, has also been documented to significantly influence outcomes, emphasizing the overarching challenge it presents to both survival and post-treatment quality of life (29, 30). A noteworthy determinant, Neutrophil Percentage, aligns with research by Gong et al., which illustrated the importance of neutrophil-related ratios, particularly Neutrophil to Lymphocyte Ratio (NLR), in influencing early neurological outcomes post-thrombolysis in stroke patients (31). Elevated neutrophil levels, often indicative of inflammation or potential infection, can exacerbate neuronal injury in ischemic brain tissue, thereby influencing outcomes. Elevated Onset Blood Sugar levels, substantiated as a potent predictor in our study, has been correlated with unfavorable recovery and heightened mortality post-stroke. Admission hyperglycemia, indicative of metabolic disruptions, predicts larger infarct size and poorer clinical outcomes in acute ischemic stroke patients by potentially impairing the blood–brain barrier and provoking excitatory chemokines release (32). Consistently, it has been integrated into various risk models forecasting adverse outcomes post-EVT (21, 25, 33). Furthermore, the Onset NIHSS Score, gauging initial stroke severity, emerges as a pivotal predictor in our analysis, aligning with the National Stroke Foundation’s emphasis on its utility in assessing stroke gravity (34). Its consistent incorporation in past research underpins its value in forecasting post-thrombectomy mortality (30).

In summary, the identified variables not only augment our knowledge concerning post-intervention prognostic trajectories but also highlight an imperative for a meticulously nuanced, patient-centric approach to treatment. Within the confines of our model, these determinants collectively present a robust predictive framework for 6-month mortality subsequent to intravascular treatment in ischemic stroke patients. Furthermore, our research proactively engages with the ongoing discourse regarding the judicious necessity and efficacy of assorted intravascular treatments, particularly in the context of patients exhibiting a heightened risk of 6-month mortality. This does not only bear substantial implications for informed clinical decision-making but also incites a critical evaluation of the true indispensability and clinical efficacy of various intravascular treatments, especially in light of patient safety and optimizing medical efficacy within a 6-month prognostic timeframe.

Therefore, our nomogram, constructed by carefully considering a spectrum of pre-treatment indicators, could surface as a robust predictive tool for estimating 6-month mortality probabilities in patients with anterior circulation stroke post-EVT. The practicality of the model is highlighted by its potential to facilitate nuanced and patient-tailored therapeutic planning, thereby navigating medical practitioners toward making informed intervention decisions and optimizing resource allocation.

A few limitations ought to be considered when interpreting the findings of this research. First, the cohort exclusively comprised patients of Asian ethnicity, thereby limiting the generalizability of our findings to other racial and ethnic populations. Second, collaborating with multiple centers undoubtedly led to a lack of standard diagnostic or treatment approaches. Nevertheless, the multicenter nature of the present study was also an advantage because it improves the generalizability of our findings. Third, the study did not incorporate imaging data, which could provide critical insights into patient pathophysiology and further refine predictive models for post-EVT outcomes in AIS patients. Despite the recognized limitations, our study encapsulates a degree of merit. The construction and validation of the nomogram were meticulously undertaken using well-curated cohorts from two distinct centers, with one center being pivotal for model development and the other fortifying external validation. Importantly, the nomogram is based on variables that are readily accessible and pertinent in real-world settings, presenting a potential, practical tool for clinicians.

In summary, the developed nomogram, utilizing variables such as Lack of Exercise, Initial TICI Score 1, MRS Score 5, Neutrophil Percentage, Onset Blood Sugar, and Onset NIHSS Score, offers a modest attempt to predict 6-month mortality probability for ischemic stroke patients following intravascular treatment. While the model leans on available pre-treatment indicators and aims to provide a practical reference for early decision-making in a clinical context, it is essential to approach its application with caution and validate its utility further within varied patient populations.

## Data availability statement

The original contributions presented in the study are included in the article/Supplementary material, further inquiries can be directed to the corresponding author.

## Ethics statement

The studies involving humans were approved by The Research Ethics Committee of Shenyang First People’s Hospital. The studies were conducted in accordance with the local legislation and institutional requirements. Written informed consent for participation was not required from the participants or the participants’ legal guardians/next of kin in accordance with the national legislation and institutional requirements.

## Author contributions

RW: Writing – original draft. MW: Data curation, Investigation, Writing – review & editing. WB: Data curation, Resources, Writing – review & editing. HZ: Investigation, Writing – review & editing. YX: Data curation, Investigation, Writing – review & editing. QH: Data curation, Investigation, Writing – review & editing. YW: Investigation, Writing – review & editing. XL: Investigation, Writing – review & editing. YS: Investigation, Writing – review & editing. ZH: Data curation, Investigation, Writing – review & editing. BX: Writing – review & editing.
