# Supplementary figures and images for: Nomogram to predict 6-month mortality in acute ischemic stroke patients treated with endovascular treatment
Source: Front Neurol. 2024 Jan 5;14:1330959. doi: 10.3389/fneur.2023.1330959 (PMC10796830; doi:10.3389/fneur.2023.1330959)

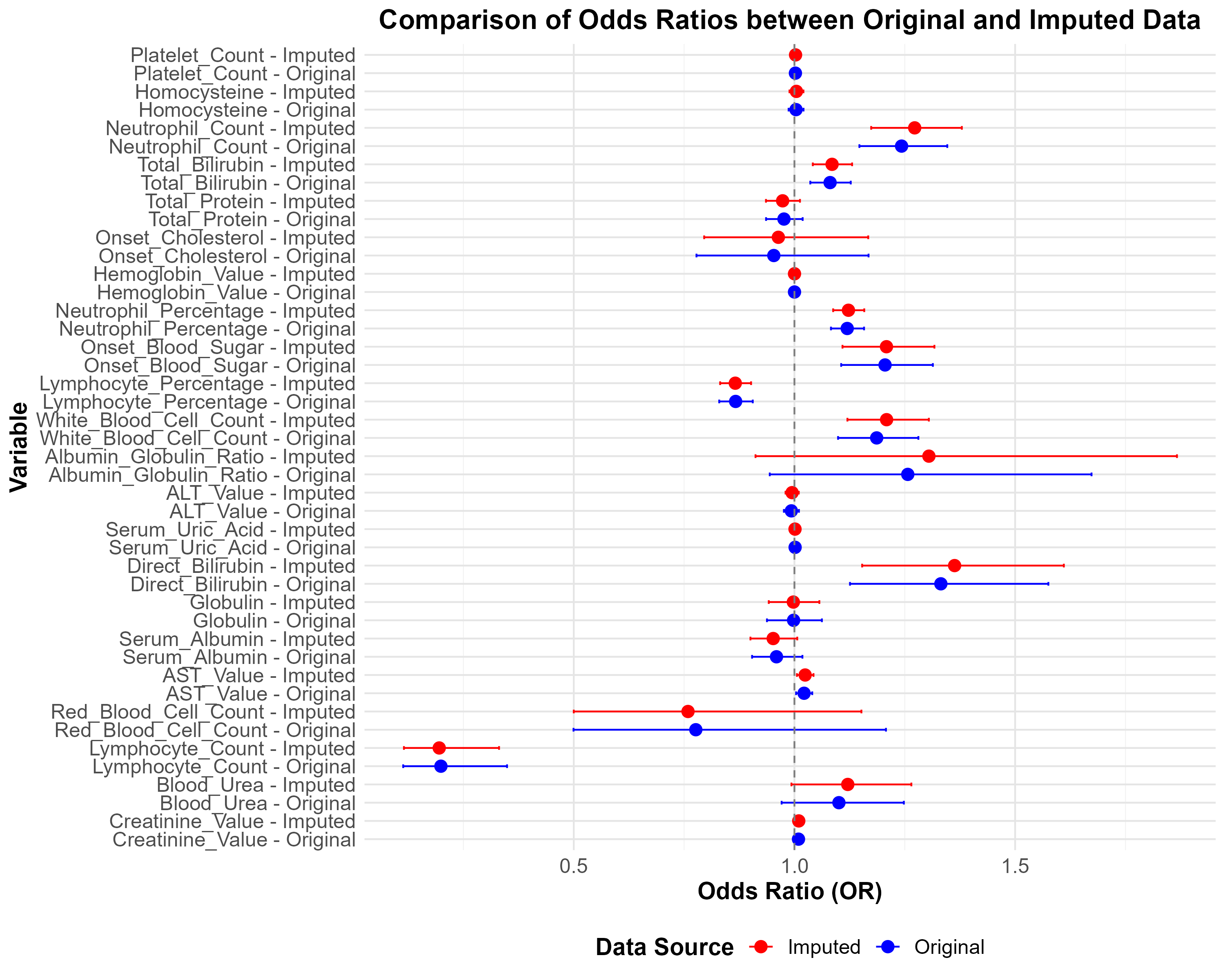

Supplement: Supplementary file 2 [file Image_1.PNG]
